# Technological Advances in Instrumental Assessment in Rehabilitation

**DOI:** 10.1155/2015/264067

**Published:** 2015-03-05

**Authors:** Giorgio Ferriero, Stefano Carda, Sasa Moslavac, Alessia Rabini

**Affiliations:** ^1^Unit of Occupational Rehabilitation & Ergonomics, Scientific Institute of Veruno, Salvatore Maugeri Foundation, IRCCS, 28010 Veruno, Italy; ^2^Department of Neuropsychology and Neurorehabilitation, University Medical Centre of Lausanne (CHUV), 1011 Lausanne, Switzerland; ^3^Spinal Unit, Special Medical Rehabilitation Hospital, 42223 Varazdinske Toplice, Croatia; ^4^Department of Gerontology, Geriatrics and Physiatrics, “A. Gemelli” University Hospital, Catholic University of the Sacred Heart, 00168 Rome, Italy

In rehabilitation research, interest in instrumental assessment is rapidly growing, particularly in the last decade. A large number of tools for instrumental assessment are now available, evaluating different aspects of the single patient or patient groups. Most of these assessment tools are disease-specific and common to other medical disciplines, for example, goniometers, and clinical tests or scales that monitor patient impairment [1]. Technological advances now make it possible to perform an in-depth evaluation of patients, analyzing their abilities across a wide range of performances. In rehabilitation, high-technology assessment tools mainly concern diagnostic devices—used to obtain outcome measurements of variables of interest—or specific equipment that is necessary to apply the tests.

In fact, in a period of increasing application of measures in clinical practice, quality control, and audit procedures, assessment has become a key process in the drive to replace the empirical approach with a scientific methodology, fundamental both to the practice of evidence-based medicine and to the strengthening of the quality of research [1]. Assessment is mainly based on a measurement process characterized by the assignment of numerical values or categories to show (according to predefined rules) the quantity of certain characteristics, functions, or behaviors.

The possibility of having an objective measurement represents a fundamental advantage in several ways; for example, it provides a scientific basis for interprofessional communication, it documents the effectiveness of treatments, and it attests their scientific credibility. Therefore, researchers are motivated to develop new instrumental assessment tools or improve old ones, demonstrating their good psychometric properties and limits. On the other hand, clinicians, who are going to use a measuring instrument, are invited to base their choice on the presence of the psychometric characteristics necessary for the specific purpose and context (preferring instruments for which the application has already been tested under conditions similar to those of interest).

Numerous scientific studies have described the main criteria for selecting an outcome measure [2, 3] and/or evaluating in detail its main psychometric properties and practices [4]. In general, the basic criterion for the choice of an instrumental assessment tool is the presence (as demonstrated through scientific publications) of adequate levels of reliability (the degree to which a measurement is free from error and, hence, the observed score gives a “true” picture), validity (degree of accuracy with which a tool measures what it is intended to measure), and responsiveness (the ability of an instrument to identify modifications or significant differences from the clinical point of view). The first two criteria are necessary for discriminative purposes (differences between subjects or groups) and predictive purposes (classification of subjects in predefined classes for prognostic purposes), while for evaluation purposes (i.e., to detect changes over time within subjects, as in the case of analysis of effectiveness of therapeutic interventions) a good level of responsiveness is also needed. Other requirements that are extremely important to consider when selecting an outcome measure are the appropriateness (degree to which the instrument responds to the questions that the specific evaluation intends to study) and accuracy (the degree to which the measuring instrument is able to capture real differences) [2, 5].

In this special issue, we invited researchers to contribute with original research articles as well as reviews investigating the benefits of instrumental assessment or to propose new technological modalities for instrumental assessment in rehabilitation.

Our aim is to stimulate researchers to publish their research in the field of technological assessment in PMR. A wide array of topics is discussed in this special issue, related to areas such as strength assessment, posture, balance and gait analysis, functional assessment tools, and cognitive and robotic assessment. Robotic devices and passive instrumented orthoses have been proposed to assess upper limb patients affected by stroke. New software for computers has been shown to improve the cognitive assessment of neurological patients, facilitating the creation of large databases and opening up new opportunities for home-based rehabilitation. Novel technological devices and assessment protocols have been demonstrated to be reliable in the evaluation of basic motor performances, in postural control, and in gait analysis.

We are edified by the large number of papers submitted and by their high scientific level.

Finally, we wish to thank not only the authors but also the expert reviewers who, with their valuable work, have made possible the publication of this special issue.

*Giorgio Ferriero* *Stefano Carda* *Sasa Moslavac* *Alessia Rabini*
